# Combination of minimum enclosing balls classifier with SVM in coal-rock recognition

**DOI:** 10.1371/journal.pone.0184834

**Published:** 2017-09-22

**Authors:** QingJun Song, HaiYan Jiang, Qinghui Song, XieGuang Zhao, Xiaoxuan Wu

**Affiliations:** 1 Tai-an School, Shandong University of Science & Technology, Tai-an, Shandong, China; 2 Department of Mechanical and Electronic Engineering, Shandong University of Science & Technology, Qingdao, Shandong, China; 3 Ji-nan School, Shandong University of Science & Technology, Ji-nan, Shandong, China; Jiangnan University, CHINA

## Abstract

Top-coal caving technology is a productive and efficient method in modern mechanized coal mining, the study of coal-rock recognition is key to realizing automation in comprehensive mechanized coal mining. In this paper we propose a new discriminant analysis framework for coal-rock recognition. In the framework, a data acquisition model with vibration and acoustic signals is designed and the caving dataset with 10 feature variables and three classes is got. And the perfect combination of feature variables can be automatically decided by using the multi-class F-score (MF-Score) feature selection. In terms of nonlinear mapping in real-world optimization problem, an effective minimum enclosing ball (MEB) algorithm plus Support vector machine (SVM) is proposed for rapid detection of coal-rock in the caving process. In particular, we illustrate how to construct MEB-SVM classifier in coal-rock recognition which exhibit inherently complex distribution data. The proposed method is examined on UCI data sets and the caving dataset, and compared with some new excellent SVM classifiers. We conduct experiments with accuracy and Friedman test for comparison of more classifiers over multiple on the UCI data sets. Experimental results demonstrate that the proposed algorithm has good robustness and generalization ability. The results of experiments on the caving dataset show the better performance which leads to a promising feature selection and multi-class recognition in coal-rock recognition.

## Introduction

Top-coal caving (TCC) is a more productive and cost-effective method compared to traditional coal mining especially in long-wall workface mining[1]. It was first applied in the 1940s in Russia and then subsequently used in France, Turkey, former Yugoslavia, Romania, Hungary, and former Czechoslovakia [2,3]. As the development of modern mining equipments, hydraulic support, conveyor, shearer and so on are widely used in coal working face [4], Coal-rock recognition(CRR) is one of the critical technique on TCC automation in fully mechanized top coal caving face [5]. Since the 1960s, more than 30 coal-rock recognition methods have been put forward, these methods covered gamma radiation, radar, vibration, infrared radiation, stress, acoustic, and so on[5–8]. MOWREY [6] developed a detecting coal interface method during the mining operation based on the continually monitor of mining machine. This approach utilized the in-seam seismic technique and adaptive learning networks to develop a seismic signal classifier for coal/roof and coal/floor interfaces detection. Based on multi-sensor data fusion technique and the fuzzy neural network, Ren, Yang and Xiong [7] put forward a coal-rock interface recognition method during the shearer cutting operation using vibration and pressure sensors. Based on Mel-frequency cepstrum coefficient (MFCC) and neural network, Xu et al. [8] proposed a coal-rock interface recognition method during top -coal caving by acoustic sensors which were fixed on the tail beam of hydraulic support. Sun and Su [5] proposed a coal-rock interface detection method for the top-coal caving face on the digital image gray level co-occurrence matrix and fisher discriminant technique. Combining image feature extraction, Hou W. [9], Reddy & Tripathy [10] gave their coal-gangue automated separation systems for the row coal in the conveyor belt transporting. Zheng et al.[11] put forward a coal-gangue pneumatic separation system for large diameter (≥50mm) coal and gangue on the basis of air-solid multiphase flow simulation by machine vision. The typical technologies of CRR can be summed up as Table 1.

**Table 1:** An overview of the typical technologies of CRR.

Technology	Principle	Limitations
γ-Rays	The detector recognize coal or rock interface using radioactive source.	The law of ray attenuation is difficult to determine, so it is difficult to recognize coal or rock.
radar	The degree of rock is detected by the speed, phase, propagation time and wave frequency of electro- magnetic wave.	When the coal thickness exceeds a certain threshold, the signal attenuation is serious, even the signal can not be collected.
vibration	Extract the coal and rock feature information of the vibration signals with signal processing techniques.	Owing to large noise disturbance, it may not be enough to derive a desired level of recognition.
infrared radiation	Identify coal or rock by the thermal distribution spectrum of shearer pick under different hardness.	Affected by environment, temperature and other factors, the detection accuracy is low.
cutting stress	By analysising the characteristics of shearer' cutting stress to identify coal or rock.	The method can’t suite to top coal caving.
acoustic	Extract the coal and rock feature information of the acoustic signals with signal processing techniques.	Affected by large noise disturbance, the detection accuracy is low.
digital image	Using image sensors, digital image processing technology and image analysis system are used to obtain the information of coal or rock.	Largely effected by dust, light and other environmental factors, the detection accuracy is low.

The shortages of the above CRR methods can be summed up as follows: (1) the application and popularization of these methods are difficulty for the environmental restriction; (2) lack of advanced and effective analytical methods for TCC; (3) the accuracies of CRR for these methods are very low for the signal interference and unnecessary energy consumption.

Since support vector machine (SVM) was proposed by Vapnik [12], it is widely used for classification in machine learning and single feature extraction, it well suites to these pattern recognition problems with small samples, nonlinearity, high dimension [13–14]. With the development of SVM theory and kernel mapping technique, many classification or regression analysis methods have been put forward. To address multi-class classification issue, Ling and Zhou[15] proposed a novel learning SVM with a tree-shaped decision frame where M/2 nodes were constructed for this model combination support vector clustering (SVC) and support vector regression (SVR). Using decision tree (DT) feature and data selection algorithms, Mohammadi and Gharehpetian [16] proposed a multi-class SVM algorithm for on-line static security assessment of the power systems, the proposed algorithm is faster and has small training time and space in comparison with the traditional machine learning methods. Tang et al. [17] presented a novel training method of SVM by using chaos particle swarm optimization (CPSO) method for the multi-class classification in the fault diagnosis of rotating machines, the precision and reliability of the fault classification results can meet the requirement of practical application.

To the problem of pattern recognition, SVM provide a new approach with a global minimum and simple geometric interpretation[13], but this method is originally designed for two-class classification[18], and has the limitation of choice of the kernel. So some new algorithms for SVM were proposed. Tsang et al.[19] gave a minimum enclosing ball (MEB) data description in computational geometry by computing the ball of minimum radius. Wang, Neskovic and Cooper [20] established a sphere-based classifier through incorporating the concept of maximal margin into the minimum bounding spheres structure. In [21], the authors extended J. Wang’s approach to multi-class problems, and proposed a maximal margin spherical-structured multi-class SVM which has the advantage of using a new parameter on controlling the number of support vectors. Using a set of proximity ball models to provide better description and proximity graph, Le et al.[22] proposed a new clustering technique which was Proximity Multi-sphere Support Vector Clustering (PMS-SVC) and was extended from the previous multi-sphere approach to support vector data description. Yildirim [23] proposed two algorithms for the problem of computing approximation to the radius of the minimum enclosing ball, both algorithms are well suited for the large-scale instances of the minimum enclosing ball problem and can compute a small core set whose size depends only on the approximation parameter. Motivated by [23], Frandi et al.[24] proposed two novel methods to build SVMs based on the Frank-Wolfe algorithm which was revisited as a fast method to approximate the solution of a MEB problem in a feature space, where data are implicitly embedded by a kernel function. Using MEB and fuzzy inference systems, Chung, Deng and Wang [25] built a Mamdani Larsen FIS (ML-FIS) SVM based on the reduced set density estimator. Liu et al.[26] proposed a multiple kernel learning approach integrating the radius of the minimum enclosing ball (MEB). In [27], the Center-Constrained Minimum Enclosing Ball (CCMEB) problem in hidden feature space of feed forward neural networks (FNN) was discussed and a novel learning algorithm called hidden-feature-space regression developed on the generalized core vector machine(HFSR-GCVM). For computing the exact minimum enclosing ball of large point sets in general dimensions, Larsson, Capannini and Källberg [28] proposed an algorithm by retrieving a well-balanced set of outliers in each linear search through the input by decomposing the space into orthants. Li, Yang, and Ding [29] proposed a novel approach for phishing Website detection based on minimum enclosing ball support vector machine, which aims at achieving high speed and accuracy for detecting phishing Website. In [30], using MEB approximation, a scalable TSK fuzzy model was given for large datasets, in the method, the large datasets were described into the core sets, the space and time complexities for training were largely reduced. Based on the improved MEB vector machine, Wang et al.[31] proposed a intelligent calculation method for traditional theoretical line losses calculation of distribution system.

It can be seen from Ref. [19] to [31], the method of MEB can improve the approximately optimal solutions and reduce time consuming. However, real-world data sets may have some distinctive distributions, generally speaking the classification problems have distinctive distributions, hence a single hyper-sphere cannot be the best description[22].

**Fig 1:**
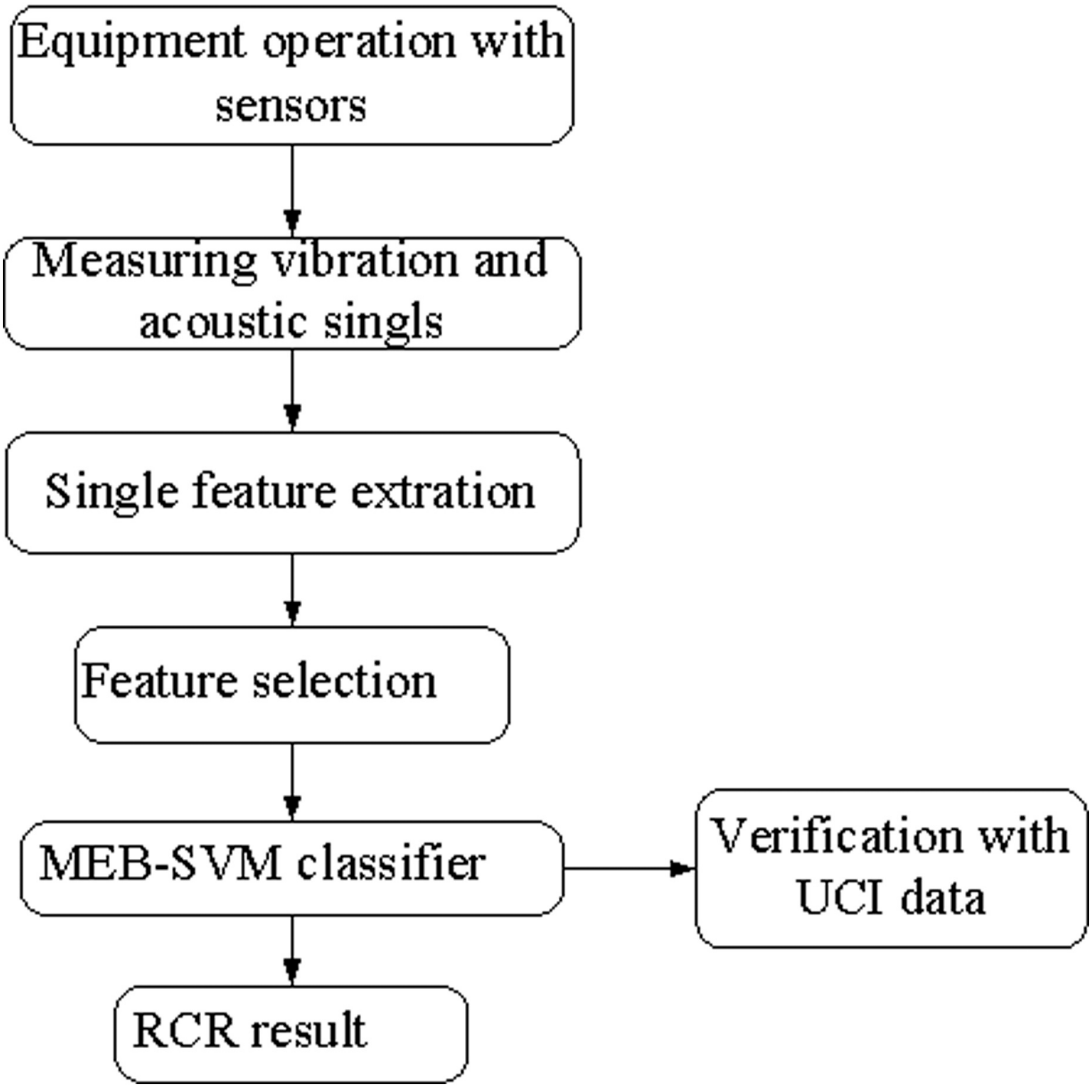
The flowchart of the study.

CRR in top-coal caving is a real-world problem, the characteristics are very complex. In this paper, we get a coal-rock(C-R) dataset with 10 feature attributes from the built acquisition model (in Section2) and propose a multi-class MEB classifier combination with SVM for CRR. The flowchart of the study is shown as Fig 1.

The rest of the paper is organized as follows: In Section 2, we designed a data acquisition model for TCC and get its real-world data set using feature construction methods. In Section 3, we put forward a multi-class SVM classifier combination with MEB and kernel trick. In Section 4, we verify our algorithm using UCI datasets with accuracy and some non-parametric tests, and carry out the method in coal-rock recognition. Finally, we make a brief conclusion in Section 5.

## Data acquisition and feature selection

### Data acquisition model

**Fig 2:**
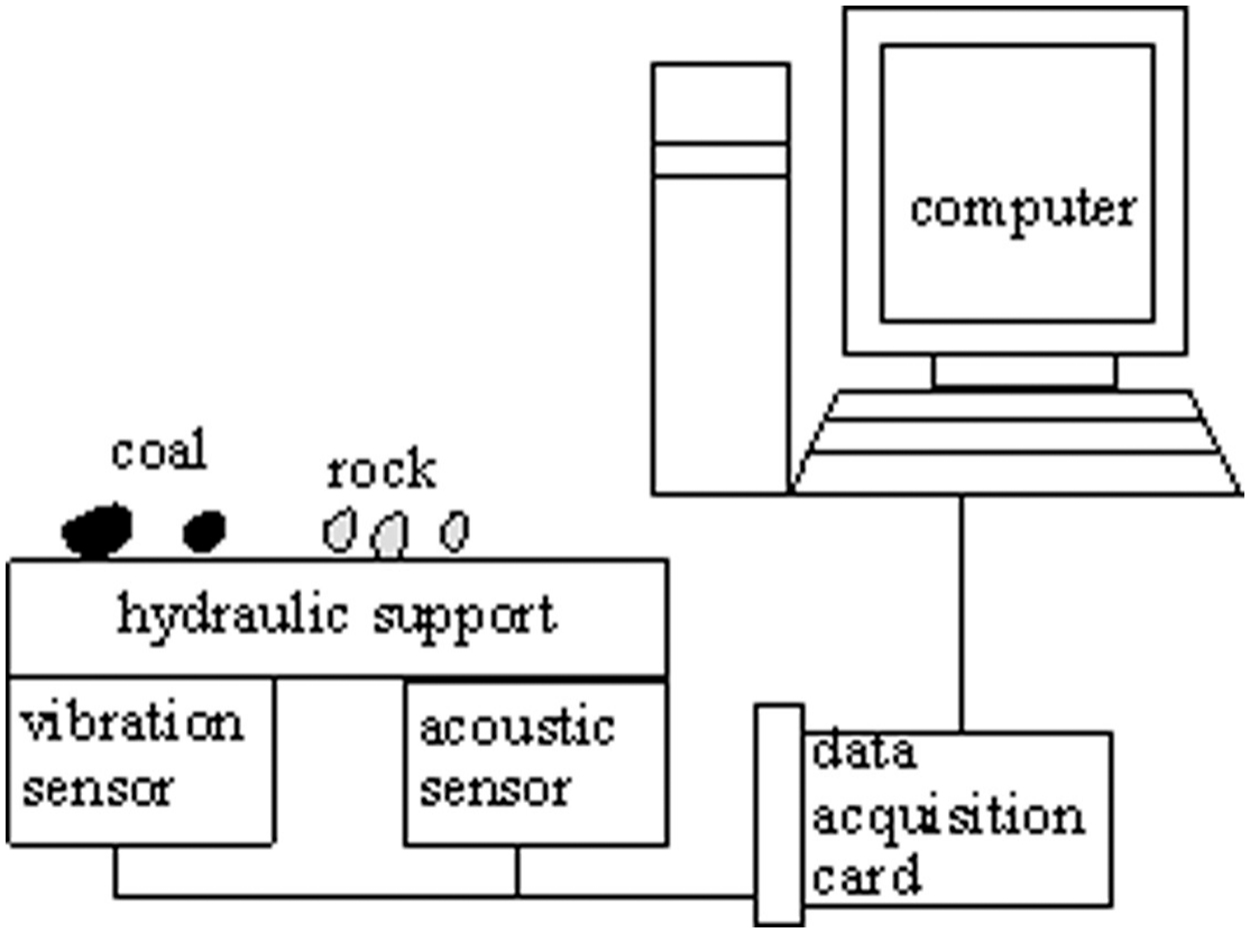
Compositions of data acquisition system for CRR.

The main purpose of this paper is to distinguish three states: whole coal, coal-rock mixture and whole rock during caving process. A series of experiment about coal-rock recognition are carried out in 11208 working face of Xinzheng coal mine, Henan Province, China. The thickness of the coal seam is between 4.5–7 meters, with an average thickness of 5 meters. The date acquisition model is shown as Fig 2.

Drawing on the experience of the above references about CRR, acoustic and vibration sensors are used to collect the caving signals. The sensors are fixed below the tail beam of hydraulic support to avoid the noise interference of conveyors and shearer in the working face. When the top coal impinges against the tail beam of the hydraulic support, sensor gets a impulse response signal which is dependent upon the state of coal-rock in the caving process. And the data are recorded using data-acquisition card PCI9810 with 8 KHz sampling frequency.

### Feature construction

The ultimate goal of pattern recognition is to well discriminate the class membership [32]. The main step of classification process on acoustic and vibration data is extraction of features from data sets. These features must contain useful information to discriminate between different objects. For vibration signal, the statistical features are usually extracted from mean, median, standard deviation, sample variance, kurtosis, skew ness, range, minimum, maximum and sum [33]. By the well-known Hilbert transforms, Huang et al.[34]in 1998 proposed a empirical mode decomposition (EMD) method for analyzing nonlinear and non-stationary data. Using the powerful time-frequency analysis technique, the complicated data set can be decomposed into a finite and often small number of intrinsic mode functions (IMFs). Through EMD, the original signals of acoustic and vibration can be decomposed into a set of stationary sub-signals in different time scales with different physical meanings[35]. So, by Hilbert-Huang transforms, the total energy (TE) of IMFs and energy spectrum entropy (ESE) of Hilbert can discriminate the characteristic of the acquired data. Fractal dimension can quantitatively describe the non-linear behavior of vibration or acoustic signal, and the classification performance of each fractal dimension can be evaluated by using SVMs [36]. Mel-frequency cepstral coefficients (MFCC) can successfully model human auditory system, and it is extensively used for speech recognition [37], so, the feature is also used in the coal-rock recognition. Discrete wavelet transform (DWT) is a time-scale analysis method, the advantage of it lies in detecting transient changes, and the total wavelet packets entropy(TWPE) measures how the normalized energies of the wavelet packets nodes are distributed in the frequency domain [38], signal energy of the wavelet transform coefficients (WTC) at each level can be separated in DWT domains, hence, TWPE can maintain an optimum time-frequency feature resolution at all frequency intervals for the vibration and acoustic signals. For vibration and acoustic signals, fractal dimension (FD) can reflect their complexity in the time domain and this complexity could vary with sudden occurrence of transient signals[39]. In this paper, general fractal dimension(GFD) of the data is calculated for the acoustic and vibration signals.

Finally, nine feature variables are selected for coal-rock recognition, they are Residual variance, Spectral Centroid, Kurtosis, Skew Ness, TE of IMFs, ESE of Hilbert, MFCC, TWPE, GFD for the two signals. Owing to acoustic and vibration two signals, there is 18 features in the C-R dataset. This section is based on our previous work[40].

### Feature selection

Recently, the amount of data typically used to perform machine learning and pattern recognition applications has rapidly increased in all areas the real-world dataset. In general, additional data and input features are thought to help classify or determine certain facts. As a result, the noise, redundancy and complexity in data have also increased, then the data that is irrelevant to other data may lead to incorrect outcomes[41]. Therefore, feature selection is necessary to remove the irrelevant input features. Feature selection can select useful features and construct a new low-dimensional space out of the original high-dimensional data. In order to optimize these feature variables and improve classification accuracy, the MF-Score(MFS) feature selection method proposed in [40] is used in this paper.

Using the evaluation criterion of feature ranking *R*(*f*_*i*_), the characteristic performance of the feature in a dataset can be gotten, *R*(*f*_*i*_) is defined as

$$R(f_{i})=\frac{\sum_{j=1}^{m}\text{D}{\left(f_{i}\right)}_{j}}{\sum_{j=1}^{m}\text{S}{\left(f_{i}\right)}_{j}}$$ (1)

where, S(*f*_*i*_) is the relative distance within the range of variance, it is defined as follows:

$$\text{S}(f_{i})=\frac{\frac{1}{n_{j}}\sum_{l=1}^{n_{j}}{\left({\left(f_{j}^{i}\right)}_{l}-{\bar{f}}_{j}^{i}\right)}^{2}-\underset{1\leq l\leq n_{j}}{\text{min}}{\left({\left(f_{j}^{i}\right)}_{l}-{\bar{f}}_{j}^{i}\right)}^{2}}{\underset{1\leq n\leq n_{j}}{\text{max}}{\left({\left(f_{j}^{i}\right)}_{l}-{\bar{f}}_{j}^{i}\right)}^{2}-\underset{1\leq l\leq n_{j}}{\text{min}}{\left({\left(f_{j}^{i}\right)}_{l}-{\bar{f}}_{j}^{i}\right)}^{2}}$$ (2)

${\left(f_{j}^{i}\right)}_{l}$ is the *l-*th sample value of classes *j* for feature *f*_*i*_ in Eq (2).

D(*f*_*i*_) is defined as an average between-class distance for feature *f*_*i*_:

$$D(f_{i})=\overset{}{\underset{1\leq j<l\leq C}{\sum }}(\frac{n_{j}+n_{l}}{N}){\left(\bar{f_{j}^{i}}-\bar{f_{l}^{i}}\right)}^{2}$$ (3)

where *N* is the number of the samples, subscripts *l* and *j* is class types, *l or j* = 1,2,…*m*. *n*_*l*_ and *n*_*j*_ represent the number of samples in classes *l* and *j*, respectively. The $\bar{f_{l}^{i}}$ and $\bar{f_{j}^{i}}$ are the means of classes *l* and *j* for feature *f*_*i*_.

*R*(*f*_*i*_) reflects how well the feature *f*_*i*_ is correlated with the class, and large value indicates strong correlation with class *i*.

After feature selection, the C-R dataset is reduced to 10 features from 18 feature variables. Table 2 shows these feature attributes of the dataset.

**Table 2:** Feature attributes of the C-R dataset after feature selection.

Feature code	Feature Meaning	signal source
F_1_	Residual variance	Acoustic signal
F_2_	TE of IMFs	Acoustic signal
F_3_	GFD	Acoustic signal
F_4_	TWPE	Acoustic signal
F_5_	Spectral Centroid	Acoustic signal
F_6_	MFCC	Acoustic signal
F_7_	Kurtosis	Vibration signal
F_8_	Residual variance	Vibration signal
F_9_	GFD	Vibration signal
F_10_	TWPE	Vibration signal

## Enclosing balls classifier with SVM

For MEB method, the feature space can be described with a minimum enclosing ball *B*_*j*_ which is characterized by its radius *R*_*j*_ and center *O*_*j*_.

$$O_{j}=\frac{1}{N_{j}}\overset{N_{j}}{\underset{i=1}{\sum }}x_{i}\;j=1,2$$ (4)

$$R_{j}=\text{max}\|x_{i}-O_{j}{\|}^{\frac{1}{2}}\;1\leq \text{i}\leq N_{j},j=1,2$$ (5)

**Fig 3:**
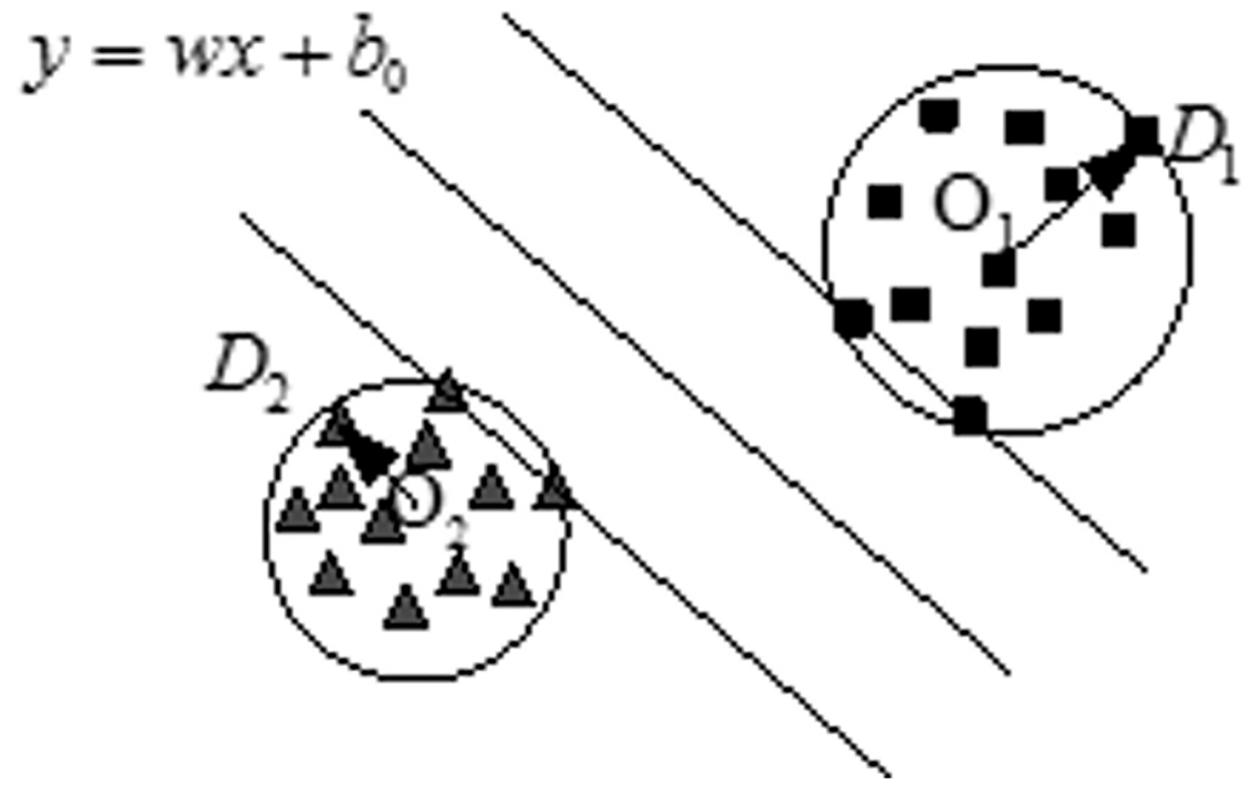
Two-class MEB-SVM classifier.

Using this method, the optimization problem can be described by Fig 3.

A multi-class MEB problem can be described as follows. Given a set of vector space *A* = {(*x*_1_,y_1_),(*x*_2_,y_2_),…,(*x*_*n*_,y_*u*_)},where, *x*_*i*_ ∈ *R*^*n*^ with *m* attributes, *y*_*j*_ ∈ {1,2,…*u*}. Using MEB, the optimization problem can be solved as follows:

$$\text{min}R_{j}{}^{2}$$ (6)

subject to

$${\left\|x_{i}-O_{j}\right\|}^{2}\leq R_{j}{}^{2}\;i=1,\ldots ,n$$ (7)

In order to take into account the samples falling outside of the balls, the slack variables *ξ*_*i*_ and regularization parameter *C* can be used in this formulas. With the soft constraints, Eqs (6) and (7) can be summarized as

$$\text{min}R_{j}{}^{2}+C\overset{n}{\underset{i=1}{\sum }}\xi_{i}$$ (8)

subject to

$${\left\|x_{i}-O_{j}\right\|}^{2}\leq R_{j}{}^{2}+\xi_{i}\;i=1,\ldots ,n$$ (9)

$$\begin{array}{ccc} C\geq 0, & \xi_{i}\geq 0 & i=1,\ldots ,n \end{array}$$ (10)

where *C* is to penalize the error samples in this EMB optimization problem, *ξ*_*i*_ is to allow the outside samples of a ball into another reasonable ball with larger radius than *R*_*j*_.

For real-world optimization problems, the samples data of a class has a high-dimensional feature space and the distribution of it is rarely spherical for its sparsity and dimensionality [19,20,26]. Generally speaking, a higher dimension is clearer to classify than a low dimension. Using a nonlinear mapping function, low-dimensional space can be transformed into higher-dimensional mapping vector space at possibly prohibitive computational cost. The basic principle of the kernel trick is to deform the lower input vector space into higher dimensional space without carrying out the function [42]. In the feature space, all patterns can be mapped into a ball when the mapping function *Φ*(*x*_*j*_) satisfies[19]:

1. the isotropic kernel (e.g. Gaussian kernel): *k*(*x*_1_,*x*_2_) = *K*(∥*x*_1_ − *x*_2_∥), or
2. the dot product kernel with normalized inputs (eg. polynomial kernel): *k*(*x*_1_,*x*_2_) = *K*(*x*_1_*x*_2_),or
3. any normalized kernel: $k(x_{1},x_{2})=K(x_{1},x_{2})/(\sqrt{K(x_{1},x_{1})}\sqrt{K(x_{2},x_{2})})$.

In this method, Gauss radial basis function is used in the kernel trick:

$$\Phi (\left\|x_{i}-O_{j}\right\|)=\text{exp}(-\frac{1}{2}{\left(\frac{\left\|x_{i}-O_{j}\right\|}{\sigma }\right)}^{2})$$ (11)

where *σ* is a width factor of the Gaussian kernel function, and it can display the points distribution of the dataset in the mapping space.

**Fig 4:**
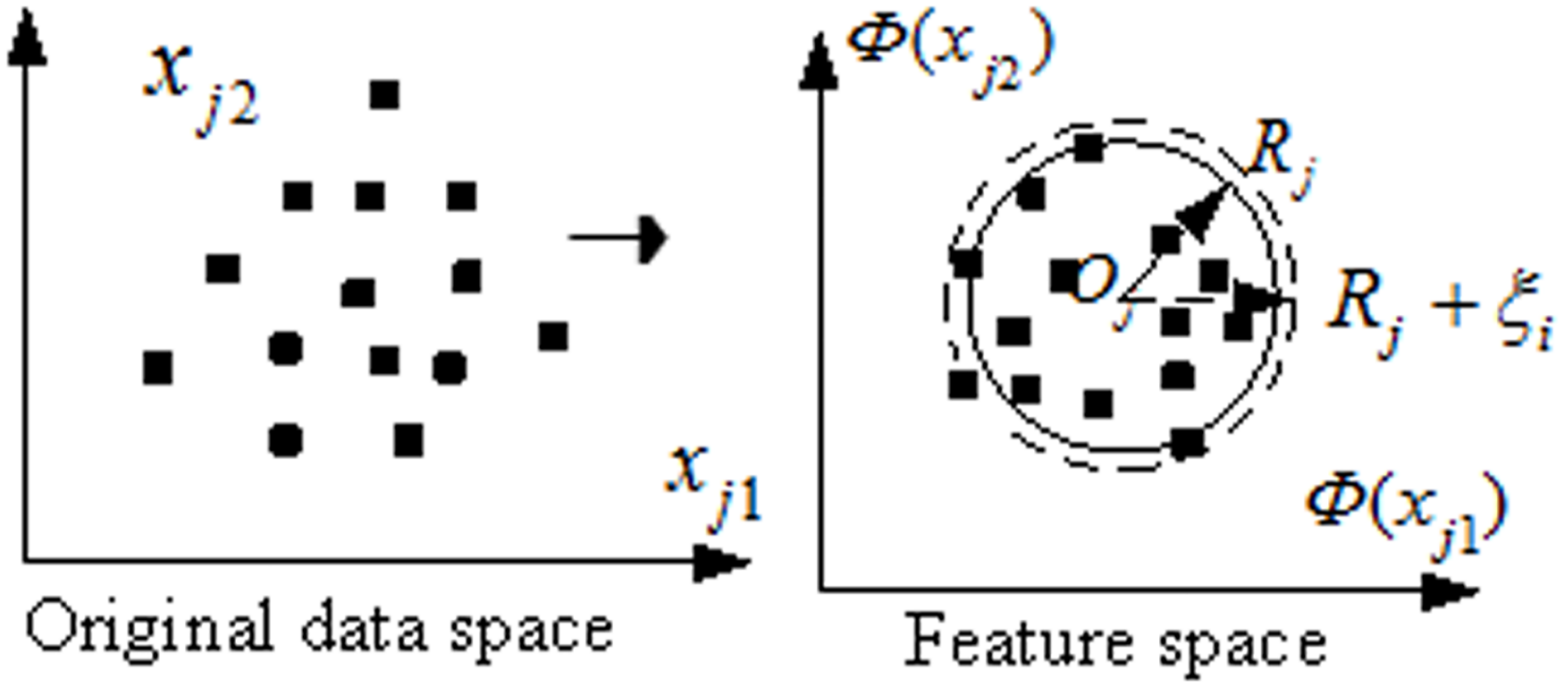
Mapping processing from input space to MED feature space (*n* = 2).

So, when the original data in the input space are mapped using kernel trick, the feature space can be transformed into a ball. Fig 4 shows the mapping processing from the input space (*n* = 2)to the mapping MED feature space using kernel functions.

For multi-class classifications problem, the purpose of MED is to find minimum enclosing balls which are characterized with radius *R*_*j*_ and center *O*_*j*_ for each class samples *x*_*j*_. Now, the radius *R*_*j*_ and center *O*_*j*_ of the MEB can be calculated in the mapping feature space as:

$$\begin{array}{l} O_{j}=\frac{1}{N_{j}}\sum_{i=1}^{N_{j}}\Phi (x_{i})=\frac{1}{N_{j}}\sqrt{{\left[\sum_{i=1}^{N_{j}}\Phi (x_{i})\right]}^{2}}\\ =\frac{1}{N_{j}}\sqrt{\sum_{\overset{i=1}{k=1}}^{N_{j}}\Phi (x_{i})\;\Phi (x_{k})}=\frac{1}{N_{j}}\sqrt{\sum_{\overset{i=1}{k=1}}^{N_{j}}k(x_{i},x_{k})} \end{array}$$ (12)

$$\begin{array}{l} R_{j}=\text{max}(\Phi (x_{i})-\frac{1}{N_{j}}\sum_{k=1}^{N_{j}}\Phi (x_{k}))=\text{max}\sqrt{{\left[\Phi (x_{i})-\frac{1}{N_{j}}\sum_{k=1}^{N_{j}}\Phi (x_{k})\right]}^{2}}\\ =\text{max}\sqrt{\Phi (x_{i})\Phi (x_{k})-\frac{2}{N_{j}}\Phi (x_{i})\sum_{k=1}^{N_{j}}\Phi (x_{k})+\frac{1}{N_{j}{}^{2}}{\left[\sum_{k=1}^{N_{j}}\Phi (x_{k})\right]}^{2}}\\ =\text{max}\sqrt{k(x_{i},x_{k})-\frac{2}{N_{j}}\sum_{k=1}^{N_{j}}k(x_{i},x_{k})+\frac{1}{N_{j}{}^{2}}\sum_{\overset{k=1}{i=1}}^{N_{j}}k(x_{i},x_{k})} \end{array}$$ (13)

Therefore, the quadratic objective function Eq (9) is represented as follows:

$${\left\|\Phi (x_{i})-O_{j}\right\|}^{2}\leq R_{j}{}^{2}+\xi_{i}\;i=1,\ldots ,n$$ (14)

In the mapping feature space, the Euclidean distance *D*_*j*_ from the sample *x*_*j*_ to the center *O*_*j*_ of the balls can be calculated as

$$D_{j}^{2}(x_{i},O_{j})={\left\|\Phi (x_{i})-O_{\text{j}}\right\|}^{2}=\;\Phi (x_{i})\cdot \Phi (x_{i})-2O_{{}_{\text{j}}}\Phi (x_{i})+{\left\|O_{\text{j}}\right\|}^{2}$$ (15)

**Fig 5:**
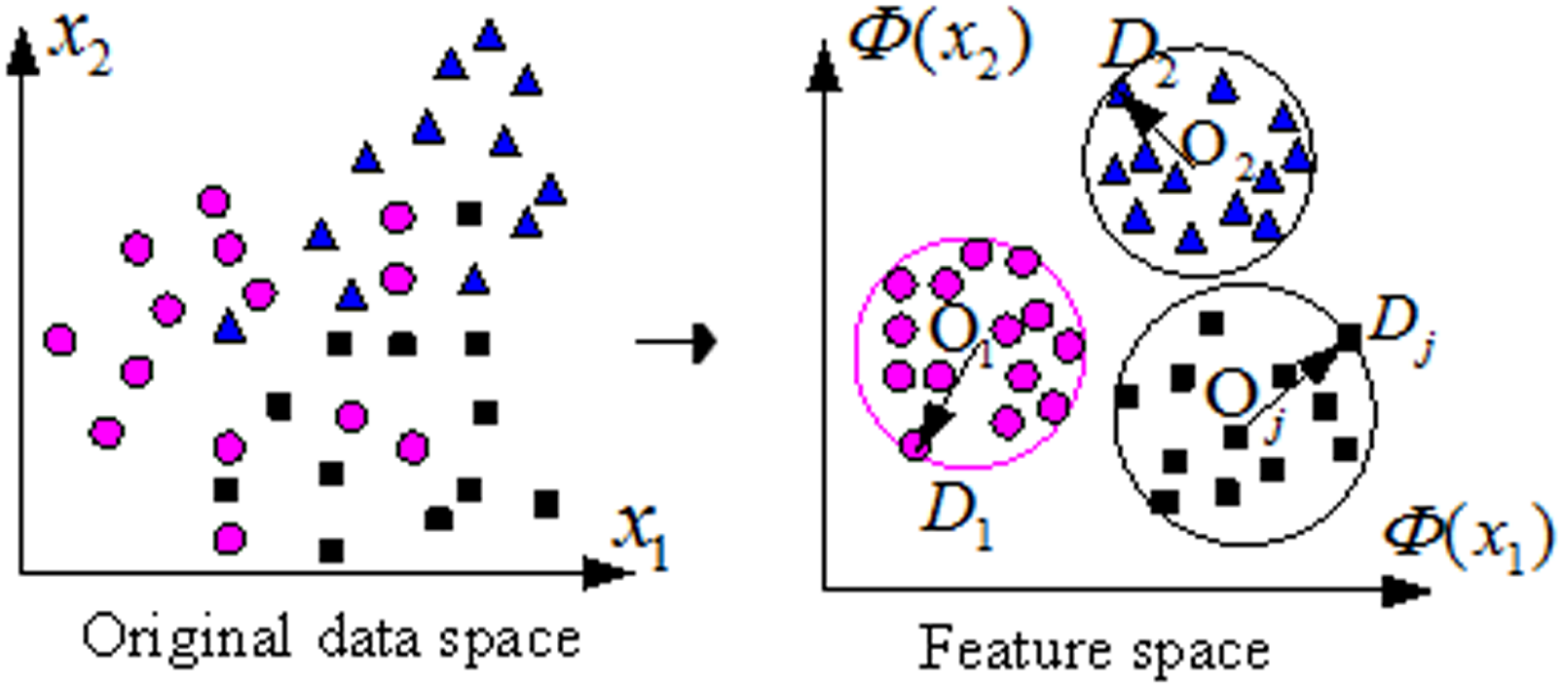
Euclidean distance in the constructed balls.

The Euclidean distance *D*_*j*_ can be explained in the constructed balls in Fig 5.

Now, the constraint condition of Eq (9) is represented as Eq (16)

$$D_{j}{}^{2}\leq {\left(R_{j}\right)}^{2}+\xi_{i}\;i=1,\ldots ,n$$ (16)

and the optimization problem is finally described as

$$\text{min}R_{j}{}^{2}+C\overset{n}{\underset{i=1}{\sum }}\xi_{i}$$

$$D_{j}{}^{2}\leq {\left(R_{j}\right)}^{2}+\xi_{i}\;i=1,\ldots ,n$$ (17)

$$\xi_{i}\geq 0\;i=1,\ldots ,n$$

The corresponding Lagrangian function for Eq (7) is determined as follows

$$L(R_{j},O_{j},\xi_{i},\alpha_{i},\beta_{i})={\left(R_{j}\right)}^{2}+C\overset{n}{\underset{i=1}{\sum }}\xi_{i}-\overset{n}{\underset{i=1}{\sum }}\alpha_{i}R_{j}{}^{2}+{\sum_{i=1}^{n}\alpha_{i}\left\|\Phi (x_{i})-O_{j}\right\|}^{2}-\sum_{i=1}^{n}\left(\alpha_{i}+\beta_{i}\right)\xi_{i}$$ (18)

where *α*_*i*_ and *β*_*i*_ are the Lagrange multipliers corresponding to each constraint.

The optimization problem becomes minimizing Eq (18) with respect to *R*_*j*_,*O*_*j*_,*ξ*_*i*_. Respectively computing these parameters’ partial derivative, and let them equal to zero, that is $\frac{\partial L}{\partial R_{j}}=0$,$\frac{\partial L}{\partial O_{j}}=0$ and $\frac{\partial L}{\partial \xi_{i}}=0$, we can get $\sum_{i=1}^{n}\alpha_{i}=1$, $O_{j}=\sum_{i=1}^{n}\alpha_{i}\Phi (x_{i})$, and 0 ≤ *α*_*i*_ ≤ *C*.

So, the above quadratic optimization problem can be formulated as following dual form

$$\text{min}(\underset{i,l:y_{i},y_{l}=j}{\sum }\alpha_{i}^{j}\alpha_{l}^{j}<\Phi (x_{i}),\Phi (x_{l})>\;-\underset{i:y_{i}=j}{\sum }\alpha_{i}^{j}<\Phi (x_{i}),\Phi (x_{i})>)$$ (19)

subject to

$$\overset{n}{\underset{i=1}{\sum }}\alpha_{{}_{i}}^{j}=1\;and\;0\leq \alpha_{{}_{i}}^{j}\leq C$$ (20)

Using Gaussian kernel function, the Euclidean distance *D*_*j*_ can be calculated as

$$\begin{array}{l} D_{j}^{2}(x_{i},O_{j})=k(x_{i},x_{i})-\sum_{j=1}^{n}\alpha_{l}^{j}k(x_{i},x_{l})+\sum_{l=1}^{n}\sum_{j=1}^{n}\alpha_{l}^{j}\alpha_{l}^{j}k(x_{l},x_{j})\\ \;=\;k(x_{i},x_{i})-\sum_{j=1}^{n}\alpha_{l}^{j}k(x_{i},x_{l})+\sum_{l=1}^{n}\sum_{j=1}^{n}\alpha_{l}^{j}\alpha_{l}^{j}k(x_{l},x_{j}) \end{array}$$ (21)

The optimization value of the multi-class classification in MEB with center *O*_*j*_ and the radius *R*_*j*_ can be summarized as

$$f_{i}(x)=\text{arg}\;\text{min}(D_{i}^{2}-R_{j}{}^{2})\;i=1,\ldots ,s$$ (22)

The above decision rule can also be redefined as

$$f_{i}(x)=\text{arg}\;\text{min}(\left(1-\frac{D_{i}{}^{2}-R_{j}^{2}}{R_{j}^{2}}\right)\;i=1,\ldots ,s.$$ (23)

## Experiments study

### Experiments for UCI data sets

In the section, some typical datasets from UCI machine learning repository(http://archive.ics.uci.edu/ml/) are employed to evaluate the classification performance of our MEB-SVM classifier. The datasets are widely used by lots of SVM research papers, they are Iris, Glass, Wine, Breast Cancer, Liver Disorders, Image Segmentation, Sonar and Waveform. Table 3 shows the details of these datasets used in the experiments.

**Table 3:** Details of the datasets from UCI repository used in the experiments.

Data sets	Abbr.	#samples	# feature variables	#class
Iris	Ir.	150	4	3
Glass	Gl.	214	9	6
Wine	Wi.	178	13	3
Breast Cancer	BC	200	30	2
Liver Disorders	LD	345	6	2
Image Segmentation	IS	2130	19	7
Sonar	So.	208	60	2
Waveform	Wa.	5000	21	3

In these used datasets, ‘Waveform’ holds 5000 samples with 3 classes and 4 feature variables, ‘Image Segmentation’ holds 2130 samples with 7 classes and 19 features,’ Sonar’ holds 208 samples with 2 classes and 60 features. From these datasets it can be seen that the sample numbers of the experiment datasets vary from 5000 (Waveform) to 150 (Iris), the class numbers of them vary from7 (Image Segmentation) to 2 (Breast Cancer, Liver Disorders and Sonar), the feature variables vary from 60 (Sonar) to 6 (Liver Disorders). In the original datasets, the class labels of the two-class datasets ‘Liver Disorders’ and ‘Breast Cancer’ datasets are ‘-1’ and ‘1’, so in the experiment datasets we changed them as ‘1’,and ‘2’ for adapting to our algorithm. For ‘Sonar’ datasets, the labels are ‘M’ and ‘R’ which mean mine and rock for the mine-rock recognition, the same change was done in the experiments.

The experiments are carried out on Intel Pentium 3.4 GHz PC with 2 GB RAM, MATLAB R2013. As a testing program, we also employed Lib-SVM program which developed by Tai-wan University Lin et al.[43] as the stranded multi-class SVM method in the experiments to compare with our method.

#### Experiments on accuracy

Demšar [44] analyzed the ICML Papers in years 1999–2003,and discovered that classification accuracy is usually still the only measure used, despite the voices from the medical and the machine learning community urging that other measures, such as AUC, should be used as well. Obviously, the classification accuracy is the most commonly used index to compare the performance of the algorithm. For achieving perfect accuracies of these datasets, k-fold cross validation [45] is used to evaluate the generalization of the classification algorithms, each dataset is divided into k subsets for cross validation. So we use 10-fold cross validation in the UCI experiments.

To verify the performance of the MFS+MEB-SVM method, we compared it to some excellent SVM classifiers proposed by other papers, they are SVM, MEB-SVM, PMS-SVC[22], DML+M+JC[45], AMS+JC[45], PSO + SVM[46], MC-SOCP [47]. We summarize the results of the comparison in Table 4.

**Table 4:** Accuracies of experiments comparing with the referenced algorithms.

Data sets	MFS+MEB-SVM	MEB-SVM	SVM	PMS-SVC	DML+M+JC	AMS+JC	PSO + SVM	MC-SOCP
Ir.	96.55	96.55	96.67	93.4	96.3	94.00	98	96.7
Gl.	82.74	75.38	72.90	81.00	69.7	81.4	78.4	73.4
Wi.	98.91	98.91	98.84	97.25	97.5	96.9	99.56	98.6
BC	88.57	88.57	90.03	98.00	96.2	94.2	97.95	80.70
LD	73.84	59.92	57.33	60.56	61.7	55.8	62.75	65.66
IS	97.43	89.65	82.43	95.83	97.3	97.9	96.53	94.4
So.	100.00	82.69	80.35	89.65	84.7	86.7	88.32	92.38
Wa.	87.80	87.80	73.52	83.9	81.8	81.9	85.00	86.6
Avg.	90.73	84.93	81.51	87.45	85.65	86.1	88.31	86.06

As can be seen from Table 4, The best method to classifying the ‘Sonar’, ‘Glass’ and ‘Liver Disorders’ data sets among all methods is the combining of MF-Score feature selection and MEB-SVM classifier, and this method obtained 100% classification accuracy on ‘Sonar’ data set. The best method to classify the ‘Iris’ and ‘Wine’ datasets is PSO+SVM. The average accuracy of MFS+MEB-SVM is much higher than that of MEB-SVM and SVM. These results have shown that the MEB-SVM has good generalization ability and the multi-class F-score feature selection method is effective and robust in the classification of the mass of datasets.

#### Experiments on non-parametric tests

The averaged results on accuracy in Table 4 show that the four algorithms(PMS-SVC, DML+M+JC, AMS+JC, MC-SOCP) have very similar predictive accuracy, that is, there is no statistical difference in accuracy between the above four algorithms. The main reason is that the accuracy measure does not consider the probability of the prediction. Based on that, we provide Friedman non-parametric statistical test for comparison of more classifiers over multiple data sets. In this section, we briefly introduce Friedman test and present an experimental study using the eight algorithms.

Friedman test is a non-parametric test equivalent of the repeated-measures ANOVA(Analysis of Variance)[48]. It ranks the algorithms for each data set separately, the best performing algorithm getting the rank of 1, the second best rank 2, and so on, In case of ties average ranks are assigned. Let $r_{i}^{j}$ be the rank of the *j*-th on the *i*-th data sets. Under the null-hypothesis, which states that all the algorithms are equivalent and so their ranks should be equal, the Friedman test compares the average ranks of algorithms, and the following defines the Friedman statistic:

The Friedman test compares the average ranks of algorithms, $R_{j}=\frac{1}{N}\sum_{i}r_{i}^{j}$.

$$\chi_{F}^{2}=\frac{12N}{k(k+1)}[\underset{j}{\sum }R_{j}^{2}-\frac{k{\left(k+1\right)}^{2}}{4}]$$ (24)

where *k* and *N* are the numbers of algorithms and data sets, respectively, and *R*_*j*_ is the average ranks of algorithms, $R_{j}=\frac{1}{N}\sum_{i}r_{i}^{j}$. When *N* and *k* are big enough the Friedman statistic is distributed according to $\chi_{F}^{2}$ with *k-1* degrees of freedom, where *N* > 10 and *k* > 5 based on experience when N and k are big enough.

The Friedman’s $\chi_{F}^{2}$is undesirably conservative, and in 1980 Iman and Davenport [49] extended this method and a better statistic is defined as:

$$F_{F}=\frac{(N-1)\chi_{F}^{2}}{N(k-1)-\chi_{F}^{2}}$$ (25)

Where *F*_*F*_ is distributed according to the F-distribution with *k−1* and *(k−1)(N−1)* degrees of freedom.

If Friedman or Iman-Davenport tests rejects the null-hypothesis, Nemenyi proceeded with a post-hoc test, which is used when all classifiers are compared to each other[50]. Then, the critical difference is calculated as follows:

$$CD=q_{\alpha }\sqrt{\frac{k(k+1)}{6N}}$$ (26)

Where *α* is significance level, *q*_*α*_ are critical values which are based on the Studentized range statistic divided by $\sqrt{2}$. The critical values are given in Table 5 for convenience.

**Table 5:** Critical values for the two-tailed Nemenyi test after the Friedman test.

#classifiers	2	3	4	5	6	7	8	9	10
*q*_0.05_	1.960	2.343	2.569	2.728	2.850	2.949	3.031	3.102	3.164
*q*_0.10_	1.645	2.052	2.291	2.459	2.589	2.693	2.780	2.855	2.920

The Bonferroni-Dunn test is a post-hoc test that can instead of the Nemenyi test when all classifiers are compared with a control classifier. The alternative way is to calculate the CD using Eq (26), but using the critical values for *a*/*(k−1)*. The critical values are given in Table 6 for convenience.

**Table 6:** Critical values for the two-tailed Bonferroni-Dunn test after the Friedman test.

#classifiers	2	3	4	5	6	7	8	9	10
*q*_0.05_	1.960	2.241	2.394	2.498	2.576	2.638	2.690	2.724	2.773
*q*_0.10_	1.645	1.960	2.128	2.241	2.326	2.394	2.450	2.498	2.539

The procedure is illustrated by the data from Table 7, which compares eight algorithms and eight data sets. The evaluating indicator of learning algorithms is AUC and the ranks in the parentheses are computed with the Friedman test in Table 7. AUC is the area under the curve of ROC(Receiver Operating Characteristic), provides a good “summary” for the performance of the ROC curves, then it is a better measure than accuracy[51]. Hand and Till[52] present a simple formula to calculating AUC of a classifier for binary classification, Huang et al. extended the formula to multi-class data sets[51].

**Table 7:** Comparison of AUC between eight algorithms.

Data sets	MFS+MEB-SVM	MEB-SVM	SVM	PMS-SVC	DML+M+JC	AMS+JC	PSO +SVM	MC-SOCP
Ir.	0.962(4)	0.971(2.5)	0.971(2.5)	0.918(8)	0.945(6)	0.921(7)	0.974(1)	0.952(5)
Gl.	0.856(1)	0.758(5)	0.758(5)	0.826(3)	0.721(8)	0.835(2)	0.751(7)	0.758(5)
Wi.	0.959(3.5)	0.951(6)	0.941(8)	0.959(3.5)	0.954(5)	0.949(7)	0.963(2)	0.969(1)
BC	0.874(7)	0.913(5)	0.897(6)	0.962(1)	0.946(3)	0.937(4)	0.951(2)	0.812(8)
LD	0.751(1)	0.652(5)	0.584(8)	0.624(6)	0.658(3.5)	0.601(7)	0.658(3.5)	0.721(2)
IS	0.978(1.5)	0.838(7)	0.815(8)	0.937(6)	0.967(3)	0.962(4)	0.978(1.5)	0.952(5)
So.	0.916(2)	0.875(4.5)	0.865(7)	0.875(4.5)	0.865(7)	0.881(3)	0.865(7)	0.941(1)
Wa.	0.853(4)	0.853(4)	0.701(8)	0.853(4)	0.828(6)	0.802(7)	0.867(2)	0.886(1)
Avg. rank	3	4.875	6.563	4.5	5.188	5.125	3.25	3.5

In this analysis, we choose MFS+MEB-SVM as the control method for being compared with the rest of algorithms, and set the significance level at 5%. If no classifier is singled out, we use the Nemenyi test for pairwise comparisons. The critical value (Table 5) is 3.031 and the corresponding *CD* is $3.031\sqrt{\frac{8\times 9}{6\times 8}}=3.712$. Since even the difference between the best and the worst performing algorithm is already smaller than that (6.563–3 = 3.563<3.712), we can conclude that the Nemenyi test is not strong enough to discover any significant differences between the algorithms.

**Fig 6:**
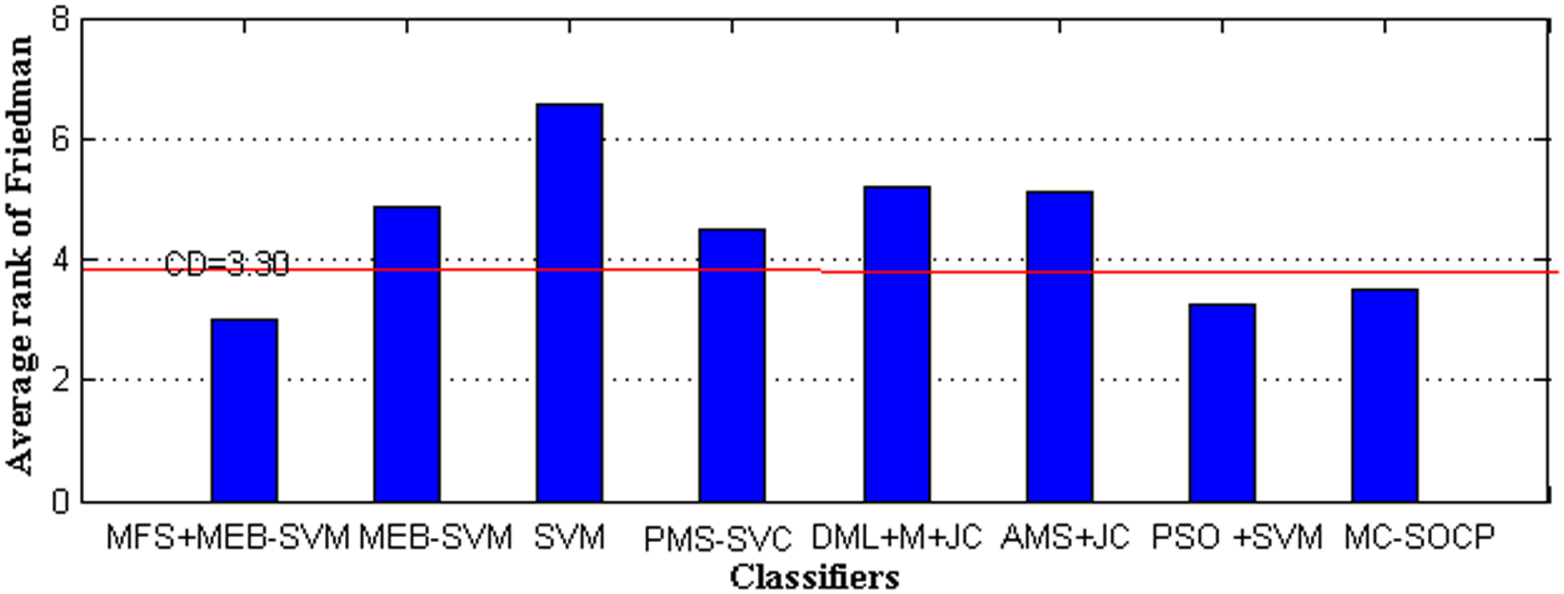
Bonferroni-Dunn test graphic.

The easiest way is to compute the CD with the Bonferroni-Dunn test. The critical value *q*_*α*_ is 2.690 for eight classifiers in Table 6, so CD is $2.690\sqrt{\frac{8\times 9}{6\times 8}}=3.30$. MFS+MEB-SVM performs significantly better than SVM (6.563–3 = 3.563>3.30). In Fig 6, we illustrate the application of Bonferroni-Dunn’s test.

This graphic represents a bar chart, whose bars have a height proportional to the average rank obtained for each algorithm by following the procedure of Friedman. A horizontal line (denoted as ‘‘CD”) is displayed along the graphic. Those bars that clearly exceed this line are the associated ones with the algorithms whose performance is significantly worse than the control algorithm. As we can see in Fig 6, the average Friedman rank of MFS+MEB-SVM is much higher than that of SVM, DML+M+JC and AMS+JC, and slightly higher than that of MEB-SVM, PMS-SVC, MC-SOCP and PSO + SVM. So, the MFS+MEB-SVM is significantly better than SVM, DML+M+JC and AMS+JC, but the difference in MEB-SVM, PMS-SVC, MC-SOCP and PSO + SVM is not significant. This indicates that MFS+MEB-SVM should be favored over SVM in machine learning and pattern recognition applications, especially when feature selection is important.

### Experiment on C-R dataset

In this section, we perform experiments on the C-R dataset which has 18 feature parameters of the acoustic and vibration signals and 1500 samples, use 10-fold cross-validation to measure the performance for consistency, and calculate the means of classification accuracy.

We first time make experiment on the subsets with single feature variable from the C-R dataset with SVM classifier. The single feature is listed in Table 2, that is, the feature selection is carried out with MF-Score. The averaged results on accuracy are shown in Table 8.

**Table 8:** Test accuracy (in %) for single feature variable subsets with MEB-SVM.

F_1_	F_2_	F_3_	F_4_	F_5_	F_6_	F_7_	F_8_	F_9_	F_10_
50.332	37.782	53.445	52.702	67.369	63.827	31.239	40.332	55.283	51.329

Table 8 shows that, in the classification of single feature variable, F_5_ (Spectrum Centroid of acoustic signal) has the highest accuracy with 67.369%, followed by F_6_ (MFCC of acoustic signal) and F_9_ (GFD of vibration signal) with 63.827% and 55.283% respectively. The other features over 50% of accuracy are F_3_, F_4_, F_1_ and F_10_, and the remaining features are under 50% in accuracy. As we see from Tables 5 and 6, it is impossible to obtain a good detection accuracy relying simply on a certain feature in the caving pattern recognition. Although Spectrum Centroid and MFCC average coefficient of acoustic signal hold the highest classification accuracy but for the vibration signal the accuracies of them are very low. This shows that a single sensor may not be enough to derive a desired level of target estimation, therefore data fusion from multiple sensors is often required.

**Fig 7:**
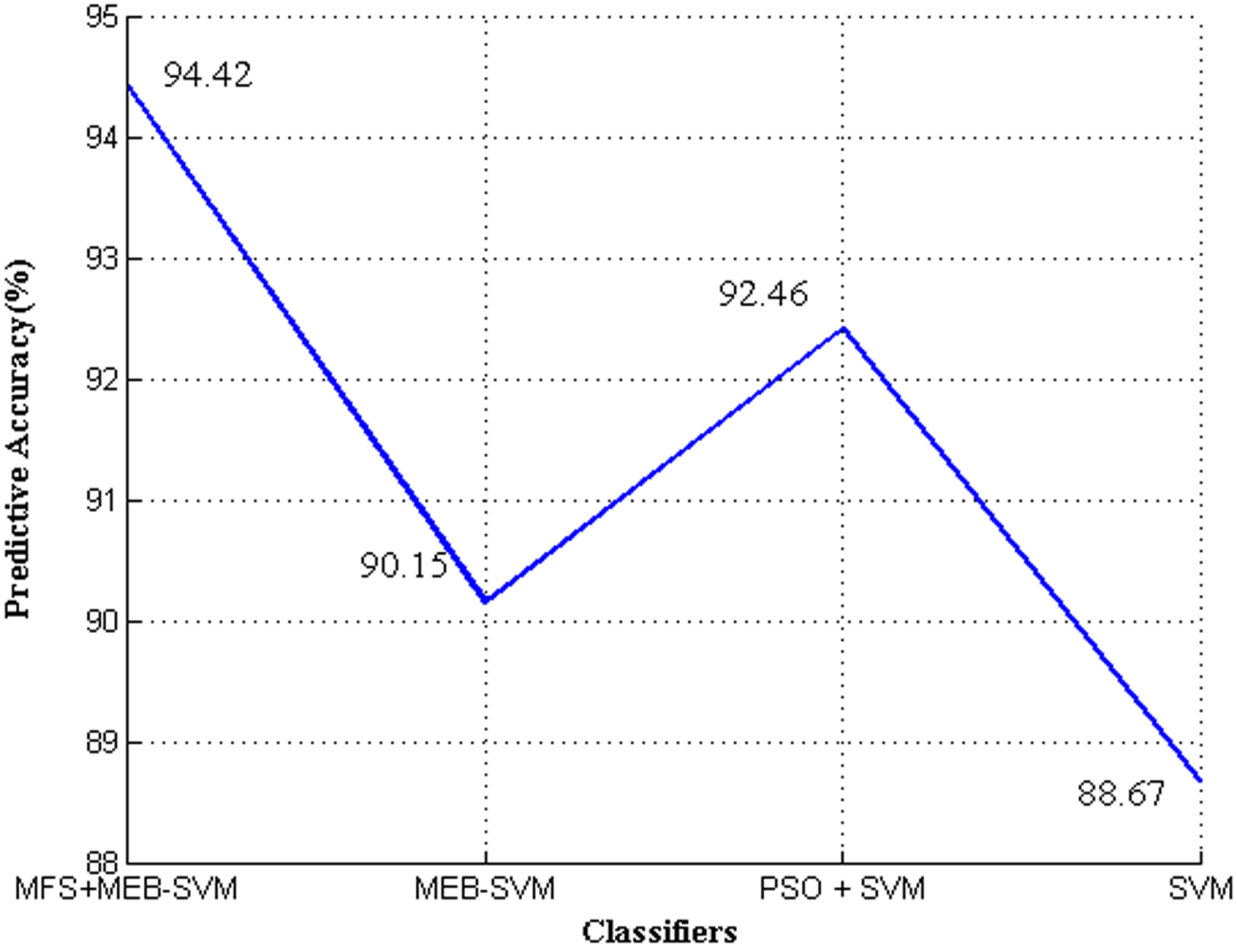
Predictive accuracy values of MFS+MEB-SVM, MEB-SVM, PSO + SVM and SVM.

Secondly, we compare accuracy of our method and to the recently developed SVM[46] and the standard SVM on the C-R data set. For the real-world data set, create 10 pairs of training and testing sets with 10-fold cross-validation and run MFS+MEB-SVM, MEB-SVM, PSO+SVM and SVM on the same training sets and test them on the same testing sets to obtain the testing accuracy. Fig 7 shows the averaged results on accuracy.

It can be seen from the comparison figure that the proposed method achieves a remarkable classification accuracy rate of 94.42% and it is superior to other methods in coal-rock recognition experiments. It is worthwhile noting that several facets should be highlighted in Fig 7. First, from the comparison of SVM and the proposed MEB-SVM, the MEB-SVM has higher recognition rates than SVM. Second, seen from the results of MEB-SVM and MFS+ MEB-SVM, the feature selection of MF-Score plays an important role, reduces the unimportant or noisy features and greatly affects the performance of classification. In addition, this MFS+MEB-SVM method may avoid over-fitting problem. Third, MFS+MEB-SVM and PSO+SVM recognition methods have similar predictive accuracies. According to empirical results, it is concluded that the proposed MFS+ MEB-SVM can help to realize the automation in fully mechanized top coal caving face.

## Conclusions

In the summary of the current research of TCC, This paper presents a recognition method of three kinds of coal-rock mixture by vibration and acoustic sensors based on MF-Score feature selection coupled with MEB-SVM classification method. We design the coal-rock data acquisition model for top-coal caving, then the C-R dataset integrated with feature construction methods of nonlinear and non-stationary data is obtained which has 18 feature attributes such as kurtosis, TE of IMFs, ESE of Hilbert, GFD, MFCC etc. Feature selection is an important task in the classification, MF-Score method is used to extract the most important feature variables and improve classification accuracy. We propose a new method of detecting coal-rock states based on minimum enclosing ball classifier with SVM, which aims at achieving high speed and high accuracy for coal-rock recognition. Through comparison with state of the art SVM methods, the experiment results illustrate the proposed MEB-SVM method has higher calculation accuracy and availability. By the designed MEB-SVM classifier, the C-R datasets is recognized with high testing accuracy more than 90 percent. On the use of non-parametric tests, we have shown a Friedman test example of performing a multiple comparison among several algorithms.

Since the proposed algorithm MEB-SVM is based on the generalized core vector machine, it is suitable for any kernel type. However, our experiments here only consider Gaussian kernel. Therefore, future work should include carrying out more experimental studies about other kernel types. What is more, analyzing the theoretical characteristics of MEB-SVM in depth and how to develop the algorithm based faster training methods for large scale datasets are also interesting topics which are our ongoing works.
